# A Study of Factors Affecting Quality of Life in Breast Cancer Patients in a Tertiary Care Centre

**DOI:** 10.7759/cureus.71738

**Published:** 2024-10-17

**Authors:** Bhushan Shah, Hariram Nivash T

**Affiliations:** 1 General Surgery, Dr. D. Y. Patil Medical College, Hospital and Research Centre, Dr. D. Y. Patil Vidyapeeth (Deemed to be University), Pune, IND

**Keywords:** assesment, breast cancer, demography, fact-b questionnaire, quality of life

## Abstract

Background

Breast cancer (BC) is one of the most common cancers among females across the globe. The impact of breast cancer and its treatment on patients' quality of life (QoL) is a significant concern. Therefore, it is critical to assess the potential underlying factors that significantly contribute to the QoL in these patients.

Objective

To determine the level of QoL of breast cancer patients and assess how QoL can impact their physical, mental, social, functional, additional well-being and their socio-demographic factors.

Methods

This cross-sectional study was conducted from August 1, 2022, to July 31, 2024, at a tertiary care centre in Maharashtra. The study included 120 women who were above 18 years of age with breast carcinoma and undergoing chemotherapy post-mastectomy. Participants were assessed using the Functional Assessment of Cancer Therapy - Breast (FACT-B) questionnaire to evaluate their quality of life. Factors like socio-demographic variables, physical, emotional, social and functional well-being were analysed by using chi-square test for the assessment of quality of life.

Results

Total of 120 women with mean age of 42.5 (SD±11.8) years were studied. Among them 61.67% were aged between 30-50 years, 94.17% of them were married 43.33% were illiterate, 51.67% had a lower socioeconomic status. FACT-B scores showed that Physical Well-Being had the lowest mean score (2.71±1.29), whereas Emotional Well-Being had the highest (1.92±1.24). Among sociodemographic factors age showed a significant association with QoL (p-0.045) while there was no significant association with marital status, educational status, or socioeconomic status.

Conclusions

The study underscores the diverse effects of BC on various aspects of QoL. The study highlights that individual patient characteristics, particularly age, may play a more prominent role in influencing QoL outcomes in BC patients.

## Introduction

Breast cancer (BC) is one of the most prevalent cancers among women worldwide. In 2020, it was the leading cause of cancer cases globally, surpassing lung cancer, with approximately 2.3 million new cases, accounting for 11.7% of all cancer cases. Epidemiological research predicts that by 2030, the number of people affected by BC worldwide will approach two million [1]. Over the past 30 years, breast cancer rates have risen globally, particularly in low- and middle-income countries. In India, breast cancer cases are projected to increase from 153,297 in 2011 to 235,490 in 2026 [2]. Projections from the World Health Organization (WHO) indicate that by the end of 2020, approximately 7.8 million women will have been diagnosed with breast cancer [3]. Improving their quality of life (QoL) is crucial for these individuals' survival.

WHO defines QoL as an individual's subjective evaluation of their overall satisfaction and well-being concerning their personal objectives, standards, aspirations, and worries, as well as the societal norms and principles that shape their existence. It is highly influenced by a person's degree of independence, mental state, physical condition, social connections, personal convictions, and proximity to salient environmental elements [4].

QoL has become an endpoint for treatment comparisons in many cancer types, especially in advanced stages. It can serve as an early warning sign of disease progression, assisting doctors in closely monitoring their patients. Numerous factors, such as functional stress, social opportunities, perceptions, impairments, and functional stress, impact QoL, which can be thought of as the impact of an illness and its treatment as experienced by patients [5].

An initial diagnosis can cause shock, fear, and disbelief, leading to both an existential and psychological crisis [6]. The side effects of cancer therapy, including chemotherapy, surgery, and radiation, coexist with the patient's chance of survival [3].

Patients with breast cancer receive various treatments, including hormonal therapy, radiotherapy, chemotherapy, and surgery. They may undergo a combination of treatments based on factors like the specific type and stage of cancer, the extent of the disease, and the patient's clinical history and demographics. Chemotherapy or hormone therapy may affect a patient's libido, fertility, and self-image, which are fundamental to femininity. According to Meneses et al., young breast cancer survivors may experience increased psychosocial distress due to potential infertility from cancer therapy toxicities, significantly impacting their QoL [4].

India's breast cancer patient survival rate is lower than that of Western countries due to factors such as early disease onset, advanced disease stage at presentation, delayed initiation of definitive management, and insufficient or fragmented treatment. The mortality rate has decreased, and the survival rate has expanded due to enhanced screening programs and medical care. As survival rates increase, QoL for these patients becomes an important component of treatment [7].

The objective of this study is to utilize the Functional Assessment of Cancer Therapy-for Breast Cancer (FACT-B) questionnaire to establish the correlation between the sociodemographic characteristics of female breast cancer patients and their QoL. The FACT-B is available in nine languages and was created with the values and conciseness of patients in mind. It is suitable for use in clinical practice and oncologic clinical trials, exhibiting validity, reliability, simplicity of administration, brevity, and adaptability to change [8].

## Materials and methods

This cross-sectional study was conducted at the Department of General Surgery, Dr. D.Y. Patil Medical College and Hospital, Pimpri, Pune. The study spanned over two years, from 1st August 2022 to 31st July 2024. Before the commencement of the study, ethical clearance was obtained from the institute’s Ethics Committee (IESC/PGS/2022/77). Informed consent was a critical component of the study, with written consent obtained from all participants.

Sample size

The sample size for this study was determined to be 120 patients. This number was selected to ensure adequate statistical power to detect significant differences and associations within the study parameters. WinPEPI software was used to calculate the sample.

Inclusion and exclusion criteria

All proven cases of breast carcinoma, women aged above 18 years, patients with recurrence of breast cancer, and those undergoing neoadjuvant chemotherapy and adjuvant chemotherapy post-modified radical mastectomy were included in the study. Male patients were excluded to maintain a focus on female breast cancer patients. Additionally, individuals in an immunocompromised state, those in critical condition, and patients with any mental or psychiatric ailments were excluded.

Data collection

Patients with biopsy-proven breast carcinoma fitting the inclusion criteria were selected for the study. Detailed histories regarding factors affecting QoL were carefully recorded using FACT-B questionnaire. These included age, educational status, marital status, and socioeconomic status. This comprehensive data collection approach helped us to understand the multifactorial nature of QoL. Data were gathered from 120 patients who had undergone breast conservation surgery or modified radical mastectomy and had completed chemotherapy.

Statistical analysis

Data collected from each patient were systematically organized and tabulated using Microsoft Excel (Microsoft, Redmond, WA, USA). Descriptive statistics were presented in the form of percentages. For further statistical analysis, GraphPad Prism 10 (GraphPad Software, La Jolla, CA, USA) was used to calculate the statistical significance. P value was calculated using the chi-squared test and values <0.05 were considered significant.

## Results

Descriptive parameters

The age of participants ranged from 30 to 50 years old, with a mean age of 42.5±11.8 years. There were 20 participants under 30 years of age, accounting for 16.67% of the total sample. The majority of participants, 74 individuals (61.67%), fell within the 30 to 50-year age group. The remaining 26 participants, representing 21.67%, were over 50 years old. Among the study population, seven (5.83%) females were unmarried, while 113 (94.17%) were married.

The educational status of the participants varied significantly in the study. Among the 120 participants, 52 individuals (43.33%), were illiterate. A smaller proportion, eight participants each, had completed primary school and intermediate or diploma education, accounting for 6.67% each. Middle school education was reported among 32 (26.67%) participants. High school education was completed by 10 participants, (8.33%), while six participants (5.00%) had graduated. Additionally, four participants, representing 3.33%, held professional qualifications.

Among the 120 participants, 62 individuals (51.67%) were classified as having lower socioeconomic status, making this the most prevalent category. The lower middle class was represented by 21 participants, or 17.50%, while 19 participants (15.83%) were upper lower class. The upper middle class had 16 participants, accounting for 13.33%, and only two participants (1.67%) were from the upper class.

Quality of life of participants

Using the FACT-B tool, female individuals diagnosed with breast cancer and undergoing chemotherapy post-mastectomy were asked to rate their QoL. The FACT-B, a validated and widely used instrument, is a 37-item self-report questionnaire designed to assess the multidimensional aspects of QoL in breast cancer patients, with a maximum score of 148. Higher scores on this scale reflect better QoL. The questionnaire includes five domains: Physical, Social/Family, Emotional, and Functional Well-Being. Apart from these, it also assesses the additional concerns. Table 1 presents a summary of the responses provided by women on the FACT-B questionnaire, detailing their experiences across these domains.

**Table 1:** Overall score of individual domains SD= Standard deviation

Order of components in the domain	Mean ± SD	Overall score (mean ± SD)
Physical		2.71 ± 1.29
Forced to spend time in bed	3.30 ± 1.22	
Feel ill	2.92 ± 1.26	
Lack of energy	2.88 ± 1.10	
Have nausea	2.66 ± 1.16	
Have pain	2.44 ± 1.30	
Bothered by treatment side effects	2.12 ± 1.18	
Trouble meeting the needs of my family	1.94 ± 1.12	
Social		2.41 ± 1.22
Accepted illness	3.26 ± 1.06	
Feel close to partner	3.06 ± 1.14	
Get emotional support from family	3.04 ± 1.24	
Feel close to friends	2.82 ± 1.32	
Get support from friends	2.53 ± 1.30	
Satisfied with family communication about Illness	2.28 ± 1.20	
Satisfied with sex life	2.21 ± 1.21	
Emotional		1.92 ± 1.24
Losing hope to fight the illness	2.58 ± 1.21	
Worry that it will get worse	2.21 ± 1.36	
Feel nervous	2.04 ± 1.11	
Satisfied with coping with illness	2.02 ± 1.17	
Worry about dying	1.75 ± 1.25	
Feel sad	1.62 ± 1.18	
Functional		2.46 ± 1.18
Accepted my illness	2.98 ± 1.10	
Content with quality of life right now	2.44 ± 1.09	
Sleeping well	2.35 ± 1.22	
Enjoying things usually do for fun	2.29 ± 1.18	
Able to enjoy life	2.21 ± 1.14	
Able to work (including work at home)	2.20 ± 1.16	
Work (including work at home) is fulfilling	1.97 ± 1.11	
Additional concerns		2.43 ± 1.26
Self-conscious about my dressing	3.21 ± 1.24	
Able to feel like a women	3.11 ± 1.10	
Bothered by weight changes	3.08 ± 1.08	
Arms are swollen or tender	2.96 ± 1.24	
Certain parts of the body where I experience pain	2.60 ± 1.42	
Been short of breath	1.94 ± 1.28	
Bothered by hair loss	1.92 ± 1.32	
Feel sexually attractive	1.83 ± 1.11	
Worry about the effect of stress of illness	1.59 ± 1.48	
Worry about that family might someday get the same illness	1.51 ± 1.50	

Physical Well-Being

In the domain of physical well-being, participants reported the lowest mean score with a mean ± SD of 2.71 ± 1.29. The specific items with the lowest scores were "use of bedtime" (2.71 ± 1.29), "feeling unwell" (2.92 ± 1.26), "insufficient energy" (2.88 ± 1.10), and "feeling queasy" (2.66 ± 1.16). Additionally, participants reported concerns about treatment side effects (2.44 ± 1.30) and difficulties providing for their families (1.94 ± 1.22). This suggests that physical well-being was notably impacted among the participants.

Social and Family Well-Being

For social and family well-being, the highest mean scores were observed for "acceptance of illness" (3.26 ± 1.06), "feeling close to partner" (3.06 ± 1.14), and "getting emotional support from family" (3.04 ± 1.24). Conversely, the lowest scores were recorded for "satisfaction with family communication about illness" (2.28 ± 1.20) and "satisfaction with sex life" (2.21 ± 1.22). The overall mean score for social/family well-being was 2.41 ± 1.22, indicating a moderate level of social support and family well-being among participants.

Emotional Well-Being

In the emotional well-being domain, participants reported the highest mean score for "losing hope in the fight against illness" (2.58 ± 1.21) and "worry that condition will get worse" (2.21 ± 1.36). Lower scores were observed for "worry about dying" (1.75 ± 1.25) and "feeling sad" (1.62 ± 1.18). The overall mean score for emotional well-being was 1.92 ± 1.24, indicating that emotional well-being was less affected compared to other domains.

Functional Well-Being

The functional well-being domain showed the highest score for "acceptance of illness" (2.98 ± 1.10), with other significant scores for "contentment with current QoL" (2.44 ± 1.09) and "sleep quality" (2.35 ± 1.22). The lowest scores were for "work fulfillment" (1.97 ± 1.11) and "ability to work" (2.20 ± 1.16). The overall mean score for functional well-being was 2.46 ± 1.18, suggesting moderate levels of functional well-being among participants.

Additional Concerns

In the additional concerns domain, the highest scores were for "self-conscious about appearance" (3.21 ± 1.24) and "feeling like a woman" (3.11 ± 1.10). Lower scores were observed for concerns such as "worry about the effect of stress of illness" (1.59 ± 1.48) and "worry that family might get the illness" (1.51 ± 1.50). The overall mean score for additional concerns was 2.43 ± 1.26, reflecting varied concerns impacting the participants’ quality of life.

The overall QoL as measured by the FACT-B questionnaire showed a mean score of 88.16 ± 17.38, reflecting a broad spectrum of patient experiences. The FACT-B domains revealed varying levels of well-being among participants: Physical well-being had a mean score of 2.71 ± 1.29, Social well-being scored 2.41 ± 1.22, Emotional well-being showed a mean of 1.92 ± 1.24, and Functional well-being had a mean of 2.46 ± 1.18. The Breast Cancer subscale, focusing specifically on concerns related to the disease, had a mean score of 2.43 ± 1.26. In terms of QoL levels, 24.17% of participants reported a poor QoL (< 50%), 55.83% reported a moderate QoL (50 - 70%), and 20% reported a good QoL (> 70%) (Table 2).

**Table 2:** Mean Scores and Distribution of Quality-of-Life Score. Data presented as n (%); n denotes the frequency

Quality of Life	Mean ± SD	
FACT-B Questionnaire Score	88.16 ± 17.38	
FACT-B Domains		
Physical well being	2.71 ± 1.29	
Social well being	2.41 ± 1.22	
Emotional well being	1.92 ± 1.24	
Functional well being	2.46 ± 1.18	
Breast cancer subscale	2.43 ± 1.26	
FACT-B Score Level	Number of participants n (%)	
< 50% (Poor)	29 (24.17)	
50 - 70% (Moderate)	67 (55.83)	
> 70% (Good)	24 (20)	

Association between QoL and demographic variables

QoL and Age 

We also assessed the association between age and QoL in women with breast cancer and found a significant and inverse correlation between age and QoL (r= -0.218, p-value=0.045). The QoL declines as one age. The association between age and QoL scores was also assessed using chi square test (Table 3).

**Table 3:** Distribution of Quality-of-Life score among different Age Groups Data presented as n (%); n denotes the frequency with p-value by chi-squared test, p value <0.05 considered statistically significant

Quality of Life Score	Age Group			Total
	< 30 Years	30 - 50 Years	> 50 Years	
< 50%	1 (5%)	22 (29.73%)	6 (23.08%)	29 (24.17%)
50 - 70%	11 (55%)	39 (52.70%)	17 (65.38%)	67 (55.83%)
> 70%	8 (40%)	13 (17.57%)	3 (11.54%)	24 (20%)
Total	20 (100%)	74 (100%)	26 (100%)	120 (100%)
P Value	0.045			

QoL and Educational Status

The association between education and QoL in women with breast cancer was evaluated using a coefficient correlation test. The results indicated a non significant correlation between education level and QoL, with a p-value of 0.63. The association between the two variables was assessed using chi square test (Table 4).

**Table 4:** Distribution of Quality-of-Life score according to Educational Status Data presented as n (%); n denotes the frequency with p-value by chi-squared test, p value <0.05 considered statistically significant

Quality of Life Score	Illiterate n(%)	Primary School n(%)	Middle School n(%)	High School n(%)	Intermediate/Diploma n(%)	Graduate n(%)	Professional n(%)	Total (%)
< 50%	15 (29%)	3 (38%)	5 (16%)	2 (20%)	2 (25%)	1 (17%)	1 (25%)	29 (24.17%)
50 - 70%	30 (58%)	4 (50%)	20 (63%)	6 (60%)	4 (50%)	2 (33%)	1 (25%)	67 (55.83%)
> 70%	7 (13%)	1 (13%)	7 (22%)	2 (20%)	2 (25%)	3 (50%)	2 (50%)	24 (20%)
Total	52 (100%)	8 (100%)	32 (100%)	10 (100%)	8 (100%)	6 (100%)	4 (100%)	120 (100%)
P value 0.63								

QoL and Marital Status

The outcomes of the chi square test indicated that the QoL scores and marital status did not significantly correlate, P value 0.52 (Table 5).

**Table 5:** Distribution of Quality-of-Life score according to Marital Status Data presented as n (%); n denotes the frequency with p-value by chi-squared test, p value <0.05 considered statistically significant

Quality of Life Score	Marital Status		Total
	Married	Unmarried	
< 50%	29 (25.66%)	0	29 (24.17%)
50 - 70%	60 (53.10%)	7 (100%)	67 (55.83%)
> 70%	24 (21.24%)	0	24 (20%)
Total	113 (100%)	7 (100%)	120 (100%)
P-Value	0.52		

QoL and Socioeconomic Status

No significant correlation was found between QoL scores and socioeconomic status, P value 0.24 (Table 6).

**Table 6:** Distribution of Quality-of-Life score according to Socioeconomic Status Data presented as n (%); n denotes the frequency with p-value by chi-squared test, p value <0.05 considered statistically significant

Quality of Life Score	Upper Class n(%)	Upper Middle n(%)	Lower Middle n(%)	Upper Lower n(%)	Lower n(%)	Total (%)
< 50%	0 (0%)	2 (13%)	5 (24%)	5 (26%)	17 (27%)	29 (24.17%)
50 - 70%	1 (50%)	9 (56%)	10 (48%)	8 (42%)	39 (63%)	67 (55.83%)
> 70%	1 (50%)	5 (31%)	6 (29%)	6 (32%)	6 (10%)	24 (20%)
Total	2 (100%)	16 (100%)	21 (100%)	19 (100%)	62 (100%)	120 (100%)
P value 0.24						

## Discussion

Evaluating QoL is a crucial aspect of clinical cancer research and trials. The degree to which a patient's anticipated level of physical, social, functional, or emotional well-being is affected by medical procedures or therapies is known as QoL. QoL is a multidimensional construct that encompasses patients' perceptions of the impact of cancer diagnosis and various treatments [9]. As survival rates improve, QoL becomes increasingly important in treatment planning. Studies underscore that QoL is a vital endpoint in clinical trials and that assessing this endpoint can enhance treatment approaches and patient satisfaction [10].

This study explored the relationship between the socio-demographic profile of female breast cancer patients post-mastectomy and their QoL. The sociodemographic profile was analyzed across four dimensions: age, marital status, educational status, and socioeconomic status. The majority of participants, 74 (61.67%), were aged between 30 and 50 years, with only 20 (16.67%) being under 30. The study found a significant association between age and QoL, aligning with the results of Yousaf et al. [4] and a study by Lu et al. [11]. However, Dehkordi et al. [12] reported no relationship between age and QoL, highlighting a discrepancy in findings.

The study also found no significant association between QoL and marital status, educational status, or socioeconomic status, which is consistent with Yousaf et al. [4] and Sharma et al. [13]. These findings suggest a need for further investigation into how factors like socioeconomic status and educational levels impact QoL, particularly in Indian women from rural areas with limited literacy. In contrast, Kanayamkandi et al. [14] found that married women had better QoL due to increased support from their spouse and children.

Lower socioeconomic status has been linked to lower QoL, as evidenced by Klein et al. [15], who found that lower income, education, and occupational status were associated with poorer QoL six months after treatment. Additionally, this study found that most women with breast cancer, 67 (55.83%), reported moderate QoL, which aligns with Yousaf et al. [4], who found a similar prevalence of moderate QoL levels.

The FACT-B questionnaire results indicated that the physical well-being domain had the lowest score among all domains, while emotional well-being had the highest. These findings are consistent with previous studies, such as Eisenbraun et al. [16], who reported poor physical, emotional, and social/family well-being. Cintamani et al. [17] also noted that family support improved the psychological status of cancer patients, with nuclear families showing higher levels of depression compared to joint families.

In terms of body image, many participants expressed dissatisfaction, which affected their social interactions. Damodar et al. [18] reported similar findings and emphasized the importance of reconstructive surgery to improve cosmetic appearance. Finally, our study revealed that QoL subscales, including the breast cancer subscale, physical well-being, and emotional well-being, were higher in patients not receiving chemotherapy, mirroring results from previous studies [19].

Limitation

Overall, our study corroborates findings from existing literature, providing insights into how socio-demographic factors influence QoL in breast cancer patients. However, this study had several limitations. Firstly, the cross-sectional design of the research restricts the ability to track changes in QoL over time and to establish causal relationships between the socio-demographic factors and QoL outcomes. Additionally, the small sample size could have introduced some. Furthermore, the FACT-B questionnaire was administered only once during treatment, without any follow-up, the study lacks data on long-term QoL outcomes.

## Conclusions

This study highlights the importance of evaluating QoL in female breast cancer patients, revealing a moderate overall QoL with significant concerns in physical well-being. The results emphasize the need for ongoing assessment of QoL, as it plays a critical role in understanding the patient experience and guiding supportive care. By examining the impact of sociodemographic factors, the study provides valuable insights that can inform targeted interventions and improve patient care. Future research should continue to explore these factors to enhance our understanding of QoL and its determinants in breast cancer patients.
